# Late-Life Inequality, CAD and Retirement Income Systems: Insights From Cross-National Analysis

**DOI:** 10.1093/geroni/igaf122.557

**Published:** 2025-12-31

**Authors:** Stephen Crystal

**Affiliations:** Rutgers University Institute for Health, New Brunswick, New Jersey, United States

## Abstract

Prior research on cumulative advantage/disadvantage in the US has shown that income inequality is higher after age 65 than at younger ages, both in within-cohort and between-cohort analyses, a pattern thought to be related to the structure of retirement income systems in the US. There has, however, been less study of late-life inequality and its relationship to inequality among non-elderly among adults across multiple countries with differing retirement income systems, particularly in the pandemic and post-pandemic period. We used OECD data to compare Gini indices of inequality for the 65+ population and the 18-64 population for 27 developed countries from 2018-2022. Results indicate that US income inequality was persistently higher at 65+ than at 18-64, with ratios ranging from 105% to 111% across years and relatively stable over the study period. In contrast, income inequality was on average lower in the other OECD countries after age 65 than before. Late-life inequality was higher in the US in each year than in any of the comparison countries, with the US inequality index (.453) exceeding the non-US rate by 36% in 2022. Countries with the lowest late-life inequality in 2022 included Czechia, Belgium, Norway, Poland, Slovenia, the Netherlands, and France, while countries with higher late-life inequality included Portugal, Chile, Israel, Latvia, New Zealand and the US. We will discuss some of the ways in which pension systems vary between countries with higher and lower late-life inequality.

